# A patient with posterior cortical atrophy due to Alzheimer’s disease

**DOI:** 10.1590/1980-57642018dn12-030015

**Published:** 2018

**Authors:** Ricardo Krause Martinez de Souza, Lea Tenenholz Grinberg, Nalini Drieli Josviak, Daniel Benzecry de Almeida, Ricardo Ramina, Pedro André Kowacs, Paulo Caramelli

**Affiliations:** 1 Instituto de Neurologia de Curitiba (INC), Curitiba, PR, Brazil.; 2 Memory and Aging Center, University of California San Francisco, USA.; 3 Universidade Federal do Paraná (UFPR), Departamento de Genética, Curitiba, PR, Brazil.; 4 Faculdade de Medicina e Hospital das Clínicas da Universidade Federal de Minas Gerais, Belo Horizonte, MG, Brazil.; 5 Departamento de Patologia, Faculdade de Medicina da USP, São Paulo, SP, Brazil.

**Keywords:** dementia, Alzheimer disease, posterior cortical atrophy, pathology, demência, doença de Alzheimer, atrofia cortical posterior, patologia

We describe a 68-year-old right-handed male with 16 years of education. He worked as a bank manager until his retirement in 1999. Ha had a past history of alcohol abuse for 19 years with no other comorbidities or family history of dementia.

Four years before the diagnosis, the patient had consulted with some ophthalmologists for visual difficulties such as reading and driving at night. He had been involved in two car accidents - neither was related to drink driving. He also presented several other minor problems driving, which might have been the first true symptoms of his illness.

The patient presented at our clinic in 2008 with progressive cognitive deficits that had begun three years earlier at the age of 57 years. The initial complaints were difficulty with calculations, mild expression difficulty (stuttering) in social situations, and occasional episodes of spatial disorientation.

**Figure 1:**
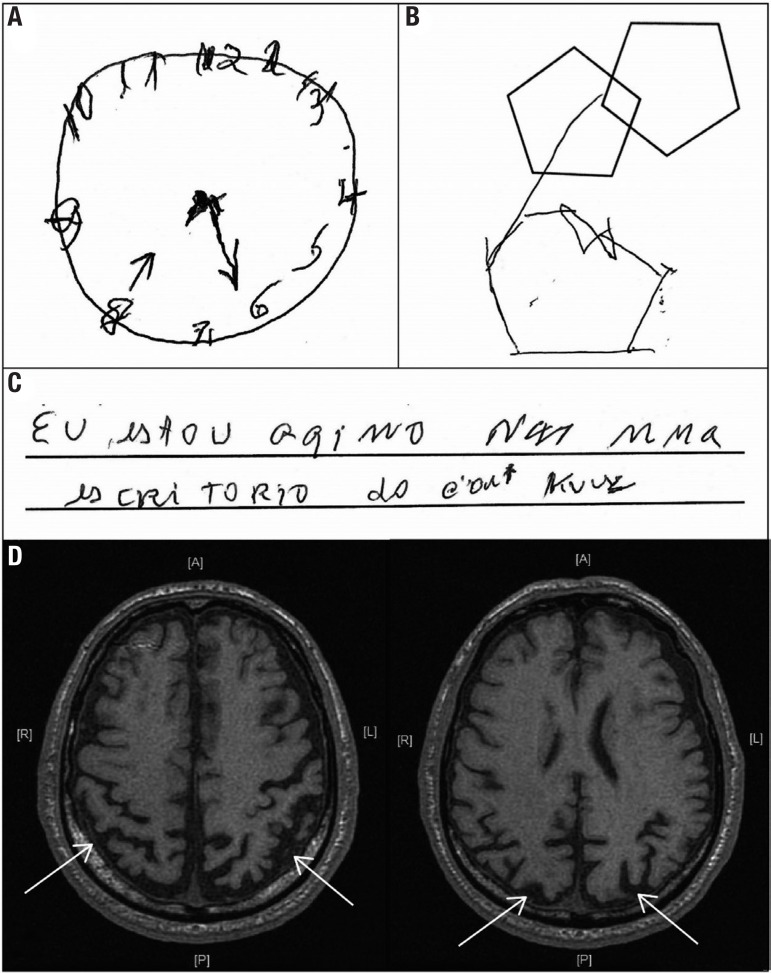
(A) Performance on clock drawing test after instruction to insert the hands of the clock to show 8:20. (B) instructions for copying the figure: showed constructional apraxia and the closing-in phenomenon. (C) In spontaneous writing with an intention to write: *“Eu estou aqui no escritório do Dr. Krause” (“I am here at the office of Dr. Krause”)*, the patient presented: repetitions of letters (mma), omitted words (aqui, Krause), inconsistent spacing between words, and mixed capital and lowercase letters (es cri TORIO). This reveals poor spatial planning on paper and slanting of the letters. (D) Magnetic resonance imaging of the brain at 1.5 T. The axial T1-weighted sequence shows marked brain atrophy of the parietal and occipital regions (white arrows).

In August 2009, he presented mild impairments of episodic memory, acalculia, visuospatial dysfunction, and constructional apraxia (Figure 1A-B). He had mild alexia and dysgraphia with slight difficulty differentiating right and left. In 2013, the patient became functionally dependent. He had moderate episodic memory deficit, disorientation in time and space, and ideomotor, dressing, and constructional apraxia. He also showed environmental agnosia, prosopagnosia, finger agnosia with severe right-left disorientation, and agraphia, (Figure 1C). In September 2013, he had generalized seizures that were controlled with lamotrigine 100 mg/day. Two years later he had to undergo a gastrostomy due to dysphagia. In 2016, the patient no longer verbalized and walked only with assistance.

The initial physical examination revealed a best-corrected visual acuity of 20/20 in both eyes. In 2013, the patient presented only slow initiating saccades and showed marked oculomotor apraxia and optic ataxia.

Laboratory testing for dementia was performed according to the recommendations of the Department of Cognitive Neurology and Aging of the Brazilian Academy of Neurology (ABN).^1^ This included a complete blood count, serum levels of creatinine and urea, free thyroxin, thyroid stimulating hormone, albumin, hepatic enzymes, vitamin B12, calcium, serological reactions for syphilis, and HIV serology. All exams were normal.

**Figure 2:**
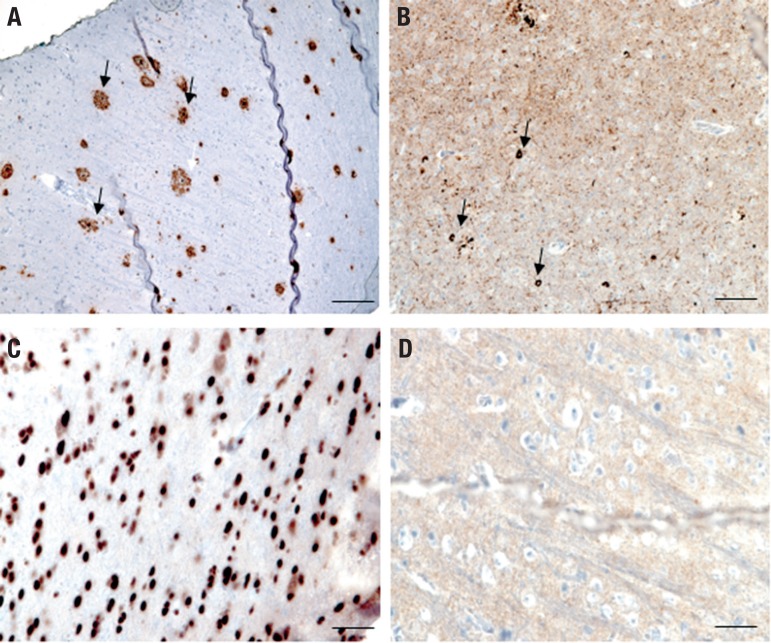
Immunohistochemistry of biopsied parietal cortex. (A) Beta-amyloid protein (4G8, 1:5000, Covance). Arrows indicate several neuritic plaques; (B) hyperphosphorylated tau (AT8, 1:500, Invitrogen). Note high density of neurofibrillary tangles (arrows) and neuropil threads; (C) TDP-43 (1:500, Proteintech). Staining is restricted to nuclei of neurons and glial cells indicating that no pathological TDP-43 inclusions were detected; (D) alpha-synuclein (LB509,1:500, Invitrogen) – negative. Scale bars: A and B – 300 μm; C and D - 50 μm.

Magnetic resonance imaging (MRI) of the brain revealed bilateral posterior cortical atrophy (Figure 1D). Although a clinical diagnosis of posterior cortical atrophy (PCA) variant Alzheimer’s disease (AD) was established, the family requested a brain biopsy to confirm this hypothesis. The biopsy findings confirmed the AD diagnosis (Figure 2).

The term PCA was coined by Benson et al. to described a series of cases with early visual dysfunction including atrophy of posterior cortical regions.^2^ PCA is a relatively rare dementia syndrome with slow and insidious evolution. The age at onset of PCA is typically 50-60 year.^3^^-^ 5 The prevalence and incidence of PCA are uncertain due to the lack of consensus among the diagnostic criteria available at different centers. However, PCA represents 4% of all dementias and 5% of Alzheimer’s disease presentations. It is more common in early-onset cases (i.e., before age 65).^6^

PCA is clinically characterized by visuospatial and visuoperceptual impairments with features of Bálint’s syndrome (simultanagnosia, oculomotor apraxia, optic ataxia) and Gerstmann’s syndrome (acalculia, agraphia, finger agnosia, left-right disorientation).^4^ Symptoms such as space perception deficit, constructional dyspraxia, environmental agnosia, dressing apraxia, alexia, limb apraxia, prosopagnosia, and visual field defect are also described in PCA.^4^^,^ 5 ^,^ 7 Other neurological features may be present including depression, hallucinations, myoclonus, limb rigidity, alien limb phenomena, tremor, and seizures.^4^^,^ 5 ^,^ 7

We describe a typical case of PCA in which the early stage shows a partial combination of both Bálint and Gerstmann syndromes. Episodic memory was relatively spared at the onset of the disease, in line with other literature reports.^3^^-^ 5 In our case, speech was slightly impaired although is often preserved at earlier stages of PCA.^4^

The MRI revealed bilateral occipito-parietal atrophy without mesial-temporal atrophy (Figure 1D). These findings are found in PCA and help to differentiate from typical AD.^4^ Several studies have shown that the pattern of cerebral atrophy is asymmetrical in PCA, with a predominance of right hemisphere involvement.^3^^,^ 4 This was not observed in our case. Histopathologic findings in PCA are often consistent with the pathology of AD,^4^ and these agreed with our findings (Figure 2). AD is the most frequent cause of PCA, accounting for about 80% of cases.^3^ Although very rare, some cases with clinical and radiological presentations of PCA have been attributed to other conditions such as dementia with Lewy bodies, corticobasal degeneration, prion disease, and subcortical gliosis.^3^^,^ 4 The objective of this case was to illustrate the main clinical and imaging characteristics of a patient with PCA, including histopathological confirmation of AD diagnosis.
